# Fingolimod—A Sphingosine-Like Molecule Inhibits Vesicle Mobility and Secretion in Astrocytes

**DOI:** 10.1002/glia.22361

**Published:** 2012-05-25

**Authors:** Saša Trkov, Matjaž Stenovec, Marko Kreft, Maja Potokar, Vladimir Parpura, Bazbek Davletov, Robert Zorec

**Affiliations:** 1 Celica d.o.o., Biomedical CenterTechnology Park 24, Ljubljana, Slovenia; 2 Faculty of Medicine, Laboratory of Neuroendocrinology-Molecular Cell Physiology, Institute of Pathophysiology, University of LjubljanaZaloška 4, Ljubljana, Slovenia; 3 Centre of excellence for Integrated Approaches in Chemistry and Biology of Proteins (CIPKeBiP)Jamova 39, Ljubljana, Slovenia; 4 CPAE, Department of Biology, Biotechnical Faculty, University of LjubljanaJamnikarjeva 101, Ljubljana, Slovenia; 5 Department of Neurobiology, University of Alabama at BirminghamBirmingham, Alabama; 6 Medical Research Council Laboratory of Molecular Biology, University of CambridgeCambridge, United Kingdom

**Keywords:** vesicle traffic, exocytosis, FTY720, sphingosine, ANP.emd

## Abstract

In the brain, astrocytes signal to the neighboring cells by the release of chemical messengers (gliotransmitters) via regulated exocytosis. Recent studies uncovered a potential role of signaling lipids in modulation of exocytosis. Hence, we investigated whether sphingosine and the structural analog fingolimod/FTY720, a recently introduced therapeutic for multiple sclerosis, affect (i) intracellular vesicle mobility and (ii) vesicle cargo discharge from cultured rat astrocytes. Distinct types of vesicles, peptidergic, glutamatergic, and endosomes/lysosomes, were fluorescently prelabeled by cell transfection with plasmids encoding atrial natriuretic peptide tagged with mutant green fluorescent protein and vesicular glutamate transporter tagged with enhanced green fluorescent protein or by LysoTracker staining, respectively. The confocal and total internal reflection fluorescence microscopies were used to monitor vesicle mobility in the cytoplasm and near the basal plasma membrane, respectively. Sphingosine and FTY720, but not the membrane impermeable lipid analogs, dose-dependently attenuated vesicle mobility in the subcellular regions studied, and significantly inhibited stimulated exocytotic peptide and glutamate release. We conclude that in astrocytes, cell permeable sphingosine-like lipids affect regulated exocytosis by attenuating vesicle mobility, thereby preventing effective vesicle access/interaction with the plasma membrane docking/release sites. © 2012 Wiley Periodicals, Inc.

## INTRODUCTION

Astrocytes, the most abundant glial cells in the human brain, provide metabolic support to neurons and participate in modulation of synaptic plasticity, learning, and memory (Perea and Araque,^2010^). Astrocytes exhibit a form of excitability, characterized by the cytoplasmic elevations in intracellular calcium (Cornell-Bell et al.,^1990^) with consequential exocytotic release of gliotransmitters (Araque et al.,^2000^; Parpura and Zorec,^2010^), such as glutamate, ATP, d-serine, and peptides, which modulate synaptic transmission (Newman,^2003^) and cerebral blood flow (Zonta et al.,^2003^), although astrocytic exocytosis continues to be debated (Agulhon et al.,^2010^; Hamilton and Attwell,^2010^).

The effectiveness of exocytotic release depends on the delivery of secretory vesicles to the plasma membrane and the multistage merger of the vesicle and the plasma membrane, leading to the cargo discharge into the exterior. The formation of the ternary SNARE (soluble *N*-ethylmaleimide-sensitive factor attachment protein receptor) complex (Jahn and Scheller,^2006^; Sutton et al.,^1998^) seems to represents an important tenant in the membrane merger and fusion pore regulation (An et al.,^2010^; Jorgacevski et al.,^2011^). Unlike in neurons and neuroendocrine cells, the SNARE complex in astrocytes consists of syntaxin-1, vesicle-associated membrane protein or synaptobrevin-2 (VAMP-2) or its homologue cellubrevin, and SNAP (synaptosomal-associated protein)-23 instead of SNAP-25 (Hepp et al.,^1999^; Parpura et al.,^1995^). Moreover, astrocytes express proteins necessary for filling vesicles with cargo molecules, such as vesicular glutamate transporters (VGLUTs) (Montana et al.,^2004^; Zhang et al.,^2004^). The assembly of SNARE complexes is further regulated by a plethora of auxiliary proteins like synaptotagmin, Rab and Sec-1/Munc-18 (Söllner,^2003^). It has also been suggested that local lipid metabolism may modulate both, cytoskeletal remodeling and exocytotic events (Janmey and Lindberg,^2004^; Rituper et al.,^2010^).

Sphingosine, a releasable constituent of membrane sphingolipids, facilitates SNARE complex assembly to enhance regulated exocytosis in neurons and neuroendocrine cells (Darios et al.,^2009^), but was never examined in astrocytes. Moreover, it is unclear whether sphingosine analogs, such as Fingolimod (FTY720; 2-amino-2-(2-[4-octylphenyl]ethyl)-1,3-propanediol) (Fujita et al.,^1994^), modulate exocytosis in astrocytes. Fingolimod, a recently introduced therapeutic for multiple sclerosis (Chun and Brinkmann,^2011^), readily crosses the blood-brain barrier (Meno-Tetang et al.,^2006^) and is phosphorylated to FTY720-phosphate (FTY720-P) predominantly by endogenous sphingosine kinase-2 (SphK-2) (Billich et al.,^2003^). FTY720-P is a structural analog of sphingosine 1-phosphate (S1P) that acts via binding to the G protein-coupled S1P receptors (S1P_1_, S1P_3-5_) (Osinde et al.,^2007^; Spiegel and Milstien,^2003^) as a high-affinity agonist (Brinkmann et al.,^2002^). Astrocytes participate in the inflammatory response in the CNS (Dong and Benveniste,^2001^), hence it is important to understand how exogenously applied sphingosine-like molecules affect astrocyte physiology; in particular because the FTY720-mediated suppression of CNS inflammation may also occur via direct action onto glial or neuronal cells (Foster et al.,^2007^).

We used confocal and total internal reflection fluorescence microscopies to investigate the impact of sphingosine-like molecules on the vesicle mobility and exocytotic discharge from astrocytes. The cell-permeable sphingosine-like molecules attenuated the mobility of peptidergic, glutamatergic, and LysoTracker-positive vesicles in astrocytes and other cells tested, and potently inhibited the release of peptides and glutamate from stimulated astrocytes, which may be important for both research and medicine.

## MATERIALS AND METHODS

### Cell Cultures and Maintenance

Primary astrocyte cultures were prepared from the cortices of 3-day-old Wistar rats (Schwartz and Wilson,^1992^). The care for experimental animals was in accordance with International Guiding Principles for Biomedical Research Involving Animals developed by the Council for International Organizations of Medical Sciences and Directive on Conditions for issue of License for Animal Experiments for Scientific Research Purposes (Official Gazette of the RS, No.40/85 and 22/87). Cells were grown in high-glucose DMEM containing 10% fetal bovine serum (Biochrom AG), 1 mM sodium pyruvate, 2 mM L-glutamine, and 25 μg/mL penicillin/streptomycin at 37°C, 5%CO_2_ and 95% air atmosphere. Subconfluent cultures were shaken at 225 rpm overnight with the subsequent medium change, which was repeated three times. Prior to experiments, the cells were trypsinized and subcultured onto poly-L-lysine-coated coverslips. In separate subsets of experiments, the cultured rat lactotrophs and mouse embryonic fibroblast–adipose-like 3T3-L1 cells were prepared as described before (Kovacic et al.,^2011^; Stenovec et al.,^2004^). Unless stated otherwise, all chemicals were purchased from Sigma-Aldrich and were of highest purity grade available.

### Cell Transfection and Vesicle Labeling

Cells were transfected with the plasmid encoding atrial natriuretic peptide tagged with emerald green fluorescent protein (ANP.emd; a gift from Dr. Ed Levitan, University of Pittsburgh, Pittsburgh, PA), using Nucleofector II (Amaxa Biosystems) and Rat Astrocyte Nucleofector Kit (Lonza) according to the manufacturer's instructions. The transfected cells were subcultured onto round 22 mm diameter poly-L-lysine-coated coverslips and observed after 40–72 h. Alternatively, astrocytes were transfected with the plasmid encoding vesicle glutamate transporter-1 fluorescently tagged with EGFP (VGLUT1-EGFP; kindly provided by Dr. Salah El Mestikawy, INSERM U513, Creteil Cedex, France) by nucleofection as described earlier. Acidic endosomes/lysosomes were stained by incubating astrocytes in the culture medium containing 200 nM LysoTracker Red DND-99 (Invitrogen) for 5 min at 37°C.

### Microscopy

To observe the mobility of ANP.emd, VGLUT1-EGFP and LysoTracker labeled vesicles, astrocyte-loaded coverslips were washed two times with extracellular solution containing 130 mM NaCl, 5 mM KCl, 2 mM CaCl_2_, 1 mM MgCl_2_, 10 mM d-glucose, and 10 mM HEPES, pH 7.2, mounted onto the recording chamber and transferred to a confocal microscope (LSM 510 & 780, Zeiss). The cells were observed by a plan-apochromatic oil-immersion objective 63×/NA 1.4. ANP.emd and VGLUT1-EGFP were excited by 488 nm argon laser line and emission fluorescence was filtered with 505–530 nm band-pass filter. LysoTracker Red DND-99 was excited with 543 nm He-Ne laser line and emission fluorescence filtered with long pass 560 nm filter. In initial experiments, the astrocyte plasma membrane was stained with FM 4-64 (2 μM, Invitrogen) dissolved in the extracellular solution, to visualize the cell perimeter. FM 4-64 was excited by 488 nm argon laser line and emission fluorescence filtered with 585 nm long-pass emission filter.

To study the mobility of fluorescently labeled vesicles, time-lapse images were acquired every 493 ms for 1–3 min in nontreated cells and cells treated with 1–20 μM FTY720 (Enzo Life Sciences), 10 μM sphingosine (Biomol), 10 μM FTY720-P (Echelon) or 10 μM thonzonium dissolved in the cell culture medium for 10 min at 37°C. In addition, exocytotic stimulus composed of L-glutamic acid (L-Glu) and ATP (1 mM final concentration, each) was applied as a bolus into the extracellular solution to all examined cells and the vesicle mobility recorded before and after stimulation.

In a separate set of experiments coverslips with ANP.emd transfected astrocytes were observed by the inverted microscope (Axio Observer.Z1, Zeiss) equipped with beam-splitter/emission-filter cubes for epifluorescence imaging, and diode-pumped solid-state (DPSS) 488 nm laser (Toptica) for TIRF imaging. The emission fluorescence was collected with α-Plan-Apochromat oil-immersion objective 100×/NA 1.46 and alternate, dual mode (TIRF and epifluorescence) images were acquired every 500 ms for 1 min prior to and 10 min after treatment with 10 μM FTY720. In epifluorescence mode ANP.emd was excited with 488 nm laser line and emission was band-pass filtered (525/31 nm). In TIRF mode ANP.emd was excited with the evanescent field formed above the coverslip glass due to the total internal reflection of 488 nm laser beam. Depending on the angle of the incident light used for TIRF imaging, the calculated (Axelrod,^2001^) penetration depth was ∼150 nm.

To determine the time-course and the relative magnitude of the *z*-axis translocation of individual vesicles imaged in the TIRF plane, we considered the exponential dependence of the TIRF excitation intensity of the fluorescent objects positioned along the *z*-axis (Axelrod,^2001^). Any change in the TIRF emission could result from: (1) changes in the vertical position of the fluorescent vesicle and/or (2) changes in the fluorescence of the vesicle itself (leak/discharge of the fluorophore, i.e., ANP.emd from vesicles, quenching/unquenching of the fluorophore, fluorophore bleaching). To assess the relative magnitude of the vesicle vertical displacement under epifluorescence illumination, we took advantage of the thin depth of field inherent to high numeric aperture objective used. The displacement of a fluorescent vesicle below or above the objective depth of field inevitably resulted in images of unacceptable sharpness and decreased vesicle fluorescence due to diminished out-of-focus emission. To monitor vesicle fluorescence at distinct *x*–*y* positions over time, we manually centered the rectangular field over the vesicle in each acquired image and assessed the average fluorescence within the field by custom-written Matlab (Math Works, Natick, MA) software t-TIME.

### Vesicle Mobility and Vesicle Discharge Studies

The mobility of peptidergic (ANP.emd), glutamatergic vesicles (VGLUT1-EGFP), and endosomes/lysomes (LysoTracker labeled) was analyzed by ParticleTR software (Celica, Ljubljana, Slovenia) in exported tiff files (Potokar et al.,^2005^). Briefly, a 2D Gaussian curve was fitted onto a selected vesicle in each image to obtain the *x*, *y* coordinates (peak of the curve), which were then connected to obtain the pathways that vesicles traveled within the total recording time. Typically, ∼50 randomly selected vesicles were tracked per cell. The mobility parameters were estimated for 15 s epochs. For each vesicle the track length (TL, the pathway that individual vesicle traveled) and maximal displacement (MD, the farthest translocation of a vesicle) were determined. The analysis of the vesicle mobility was performed in nontreated cells and in cells either acutely treated with 10 μM FTY720, or pretreated with 1–20 μM FTY720, 10 μM sphingosine, 10 μM FTY720-P, or 10 μM thonzonium for 10 min. The mean (±s.e.) vesicle TL and MD were determined in 5–15 nontreated and treated cells.

The exocytotic cargo release from ANP.emd transfected astrocytes was determined in time-lapse images by two different approaches. Individual vesicle fusions with the plasma membrane followed by ANP release were identified as a sudden decrease in the vesicle fluorescence indicating cargo discharge (Stenovec et al.,^2004^). The time-resolved fluorescence changes at the place of individual secreting vesicles were obtained by the LSM 510 and 780 software (Zeiss). The overall efficiency of vesicle cargo discharge following particular cell treatment was estimated by counting vesicles in exported tiff images using ImageJ software. The vesicles were counted on three consecutive confocal images taken before and 1 min after stimulation and the mean number of discharged vesicles determined relatively to their initial number. To test whether FTY720 alone stimulates exocytotic cargo discharge, the resting cells were observed for 1 min and then acutely treated with 20 μM FTY720 for the following 10 min. To test whether the cell pre-treatment with lipid compounds affects the vesicle cargo discharge, cells were pretreated with 20 μM FTY720, 10 μM sphingosine, or 10 μM thonzonium at 37°C for 10 min and subsequently exposed to the exocytotic stimulation by 1 mM L-Glu and 1 mM ATP. For statistical evaluation the vesicle numbers before and after stimulation were counted in 4–10 cells. The efficacy of exocytosis was expressed as the mean fraction of discharged vesicles after stimulation in nontreated and pretreated cells.

### Glutamate Measurements

The release of glutamate from confluent cultures was measured fluorimetrically (Innocenti et al.,^2000^) by epifluorescence microscope (Zeiss) using Polychrome V illumination (Till Photonics). The 360 nm excitation light was delivered to the cells via the LCI plan-neofluar water-immersion objective 63×/1.3 and NADH emission band-pass filtered (445/50 nm). Cells were bathed in extracellular solution supplemented with L-glutamic dehydrogenase (GDH; 78 U/mL) and 1 mM β-nicotinamide adenine dinucleotide (NAD^+^). Any glutamate release from nontreated and FTY720 treated cells stimulated by 1 mM ATP was detected as an increase in NADH fluorescence. Time-lapse images were taken at 2 s intervals (250 ms exposure time) by Andor Clara camera. The circular regions (2*r* = 5 μm) were positioned over the imaged cells by Till Offline Analysis software to assess NADH fluorescence increase and the fluorescence peak (in A.U.) measured.

### Statistical Analysis

The parameters of NADH fluorescence, vesicle mobility, cell and vesicle counts were expressed as the mean ± s.e. Statistical significance was determined with the two-tailed Student's *t*-test.

## RESULTS

### Dose-dependent Vesicle Mobility Attenuation by FTY720

**Fig. 1:**
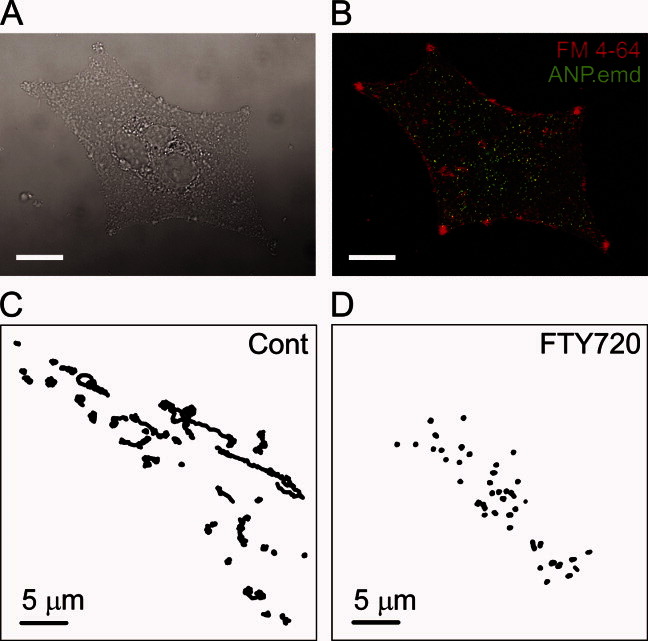
The mobility of astrocytic peptidergic vesicles is reduced by FTY720. (**A**) Live cultured astrocyte under DIC optics and (**B**) the confocal image of the same cell transfected to express ANP.emd that is stored inside peptidergic vesicles observed as numerous green fluorescent puncta; scale bars, 20 μm. (**C**) Vesicle tracks (*n* = 40) obtained in nontreated control astrocyte (Cont) and in (**D**) another astrocyte treated with 20 μM FTY720. Many elongated vesicles tracks in the control cell indicate substantial vesicle mobility. In the cell treated with 20 μM FTY720, the mobility of vesicles was attenuated; note the absence of elongated vesicle tracks. [Color figure can be viewed in the online issue, which is available at wileyonlinelibrary.com.]

To investigate the effect of FTY720 treatment on vesicle mobility, the fluorescently labeled peptidergic vesicles preloaded with ANP.emd (Han et al.,^1999^; Krzan et al.,^2003^) were monitored in cultured astrocytes (Fig. 1A) by confocal microscopy and the motion patterns of individual vesicles (Fig. 1B) analyzed. The vesicle pathways (tracks) obtained in nontreated cells were elongated (Fig. 1C), indicating substantial mobility, while in FTY720 treated cells vesicle tracks were highly contorted (Fig. 1D), indicating strongly attenuated vesicle mobility.

**Fig. 2:**
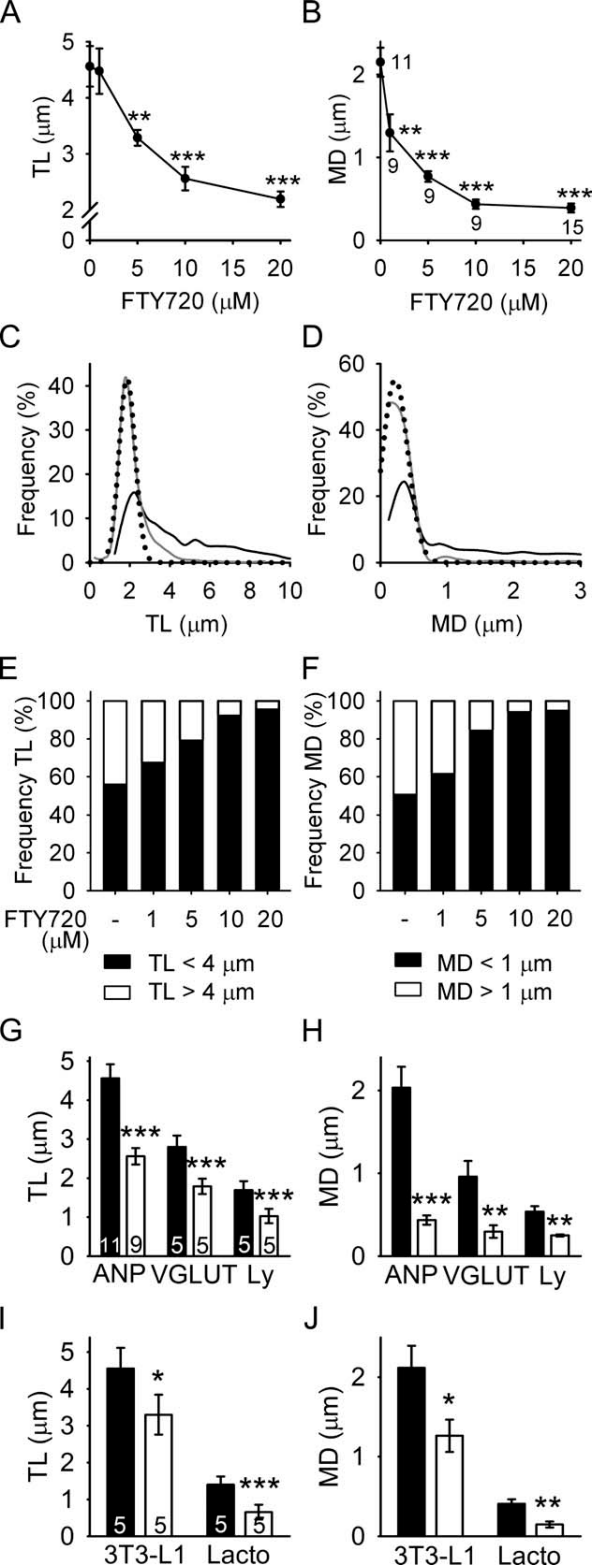
Dose-dependent attenuation of vesicle mobility by FTY720. (**A**) The mean (±s.e.) TL and (**B**) MD of peptidergic vesicles in nontreated cells and in cells treated with 1, 5, 10, and 20 μM FTY720. Already 1 μM FTY720 significantly reduced vesicle MD. At higher FTY720 concentrations (>5 μM), MD and TL were significantly reduced. Numbers below the data points indicate the number of cells analyzed. **P < 0.01, ***P < 0.001 versus nontreated controls. (**C**) The frequency distribution of vesicle TL and (**D**) MD determined in nontreated controls (black) and in cells treated with 20 μM FTY720 (gray). The TL and MD distributions in cells treated with 20 μM FTY720 were fitted best with the Gaussian functions (dotted line) *f* = *a* × exp(−0.5 − ((*x* – *x*_0_)/*b*)^2^) described in results. (**E**) Relative proportion of less (TL < 4 μm, black) and more mobile (TL > 4 μm, white) vesicles in controls and in cells treated with the increasing FTY720 concentrations. (**F**) Relative proportion of less displacing (MD < 1 μm, black) and more displacing (MD > 1 μm, white) vesicles in controls and in cells treated with the increasing FTY720 concentrations. (**G**) The TL and (**H**) MD for ANP.emd (ANP), VGLUT1-EGFP (VGLUT), and LysoTracker (Ly) labeled vesicles in nontreated cells (black bars) and in cells treated with 10 μM FTY720 (white bars). Numbers at the bar bases indicate the number of cells analyzed. Note significant reduction in TL and MD in all three vesicle types examined in astrocytes treated with FTY720. ***P < 0.001, **P < 0.01 (two-tailed paired *t*-test for VGLUT and Ly vesicles). (**I**) The mean (± s.e.) TL and (**J**) MD for ANP.emd vesicles in nontreated (black bars) 3T3-L1 cells (3T3) and cultured lactotrophs (Lacto) and the respective cells treated with 10 μM FTY720 (white bars). Note that FTY720 treatment significantly reduced vesicle mobility in the additional two cell types. ***P < 0.001, **P < 0.01, *P < 0.05 (two-tailed paired *t*-test).

To quantify the differences in vesicle mobility, two parameters—the track length (TL) and the maximal displacement (MD) were determined for each tracked vesicle within 15 s. In total, the mobility of vesicles (*n* = 1385–2916) derived from 9 to 15 cells exposed to control conditions and to different FTY720 concentrations (1, 5, 10, and 20 μM) was analyzed. The mean vesicle TL in nontreated cells was 4.56 ± 0.36 μm (*n* = 11), while in FTY720 treated cells the TL decreased in a dose-dependent manner from 4.47 ± 0.40 μm (n = 9) to 3.28 ± 0.14 μm (*n* = 9), 2.56 ± 0.21 μm (*n* = 9), and 2.19 ± 0.14 μm (*n* = 15) for 1, 5, 10, and 20 μM FTY720, respectively (Fig. 2A). Except for 1 μM FTY720 (*P* = 0.877), the TL in FTY720 treated cells diminished significantly when compared with nontreated cells (*P* < 0.01 for 5 μM FTY720, *P* < 0.001 for 10 and 20 μM FTY720). The MD significantly (*P* < 0.01 for 1 μM FTY720 and *P* < 0.001 for higher concentrations) diminished in a dose-dependent fashion from 2.15 ± 0.18 μm in nontreated cells to 1.30 ± 0.22 μm, 0.77 ± 0.06 μm, 0.44 ± 0.06 μm, and 0.39 ± 0.05 μm in cells treated with 1, 5, 10, and 20 μM FTY720, respectively (Fig. 2B). These results demonstrate that FTY720 treatment reduced vesicle mobility in astrocytes (Fig. 2A,B); however the relative attenuation was stronger for MD (81.9% at 20 μM FTY720) than for TL (51.9% at 20 μM).

The TL and MD frequency distribution plots constructed for nontreated cells and cells treated with 20 μM FTY720, revealed further important differences. In nontreated cells the vesicle TL was asymmetrically distributed (Fig. 2C, black curve) and ranged from 1.25 to 24.75 μm with the modal value of 2.25 μm (15.8%). In FTY720 (20 μM) treated cells, the majority of vesicles traveled far less, which resulted in diminished TL distributed symmetrically around the modal value of 1.75 μm (41.7%) (Fig. 2C, gray curve). A similar pattern was observed for MD, where vesicles in nontreated cells displaced a variable distance ranging from 0.12 μm up to 13.88 μm with the modal value of 0.37 μm (24.2 %), while in FTY720 treated cells, the majority of vesicles displaced only a short distance, with the modal value of around 0.12 μm (48.9%) (Fig. 2D, black and gray curve). The TL and MD frequency distribution data obtained in cells treated with 20 μM FTY720 were fitted best with the Gaussian function of the form *f* = *a* × exp(−0.5 × ((*x* − *x*_0_)/*b*)^2^), whereby for the TL plot the parameters were *a* = 41.63 ± 2.28, *b* = 0.40 ± 0.02 μm, *x*_0_ = 1.87 ± 0.03 μm and for the MD plot *a* = 55.22 ± 0.58, *b* = 0.19 ± 0.00 μm, *x*_0_ = 0.22 ± 0.00 μm (Fig. 2C,D). On the basis of the fitted functions we determined the boundary values, which were used to classify vesicles either as more (TL > 4 μm) or less mobile (TL < 4 μm), and displacing more (MD > 1 μm) or less (MD < 1 μm), consistent with the vesicle classification reported previously (Potokar et al.,^2005^). The fraction of less mobile vesicles (TL < 4 μm) increased in a dose-dependent manner from 56.3% in nontreated controls to 67.9%, 79.6%, 92.8%, and 96.0% in cells treated with 1, 5, 10, and 20 μM FTY720, respectively (Fig. 2E). Similarly, the fraction of less displacing vesicles (MD < 1 μm) increased from 51.0% in controls to 62.0%, 84.9%, 94.7%, and 95.4% (Fig. 2F) in FTY720 treated cells. This conversely implies that the treatment with 20 μM FTY720 caused more than 10-fold decrease in the number of mobile (TL > 4 μm) and more displacing (MD > 1 μm) vesicles when compared with nontreated cells.

### FTY720 Attenuates Mobility of Distinct Vesicle Types

Next, we examined whether FTY720 also affects the mobility of glutamatergic vesicles (VGLUT1-EGFP) and acidic vesicles (endosomes/lysosomes) labeled by LysoTracker in addition to peptidergic vesicles. As observed before, the mobility of different vesicle types in nontreated astrocytes varied substantially. The ANP vesicles were the fastest vesicles observed (0.31 ± 0.02 μm/s), followed by VGLUT1 (0.19 ± 0.02 μm/s) and then by LysoTracker-labeled vesicles (0.11 ± 0.02 μm/s) that were nearly three times slower than the ANP vesicles. These results are in agreement with the previous reports (Potokar et al.,^2005^, ^2010^). The average TL of ANP, VGLUT-1, and LysoTracker labeled vesicles was 4.56 ± 0.36 μm (*n* = 11), 2.80 ± 0.30 μm (*n* = 5), and 1.69 ± 0.23 μm (*n* = 5), respectively (Fig. 2G, black bars), while the respective average MD was 2.03 ± 0.25 μm, 0.96 ± 0.19 μm, and 0.54 ± 0.07 (Fig. 2H, black bars). The treatment of astrocytes with 10 μM FTY720 for 10 min significantly attenuated the mobility of all three vesicle types, reducing the TL by ∼44% (2.6 ± 0.21 μm; *n* = 9; *P* < 0.001), ∼36% (1.79 ± 0.20 μm; *n* = 5; *P* < 0.001), and ∼39% (1.03 ± 0.18 μm; *n* = 5; *P* < 0.001) (Fig. 2G, white bars) and the MD (Fig. 2H, white bars) by ∼79% (0.44 ± 0.06 μm; *P* < 0.001), ∼69% (0.29 ± 0.08 μm; *P* < 0.01), and ∼54% (0.25±0.01 μm; *P* < 0.05) for ANP, VGLUT-1, and LysoTracker-labeled vesicles, respectively.

Furthermore, we examined whether FTY720 affects the mobility of ANP.emd-loaded vesicles in additional two cell types—rat lactotrophs and mouse embryonic fibroblast-adipose-like 3T3-L1 cells. Although the same vesicle type was observed, the spontaneous mobility substantially differed in resting 3T3-L1 cells and cultured lactotrophs (TL: 4.55 ± 0.56 μm vs. 1.40 ± 0.22 μm and MD: 2.12 ± 0.28 μm vs. 0.41 ± 0.06 μm, black bars, Fig. 2I–J). The 10-min treatment of the same cells with 10 μM FTY720 significantly attenuated the vesicle mobility, reducing their TL by ∼27% (3.30 ± 0.54 μm; *n* = 5; *P* < 0.05) and ∼53% (0.65 ± 0.20 μm; *n* = 5; *P* < 0.001) (Fig. 2I, white bars) and the MD (Fig. 2H, white bars) by ∼40% (1.26 ± 0.20 μm; *P* < 0.05) and ∼64% (0.15 ± 0.04 μm; *P* < 0.01) in 3T3-L1 and lactotrophs, respectively. Our results thus suggest that FTY720 acts as a general mobility attenuating agent affecting various vesicles in three different cell types.

### Sphingosine Attenuates Vesicle Mobility in Astrocytes

**Fig. 3:**
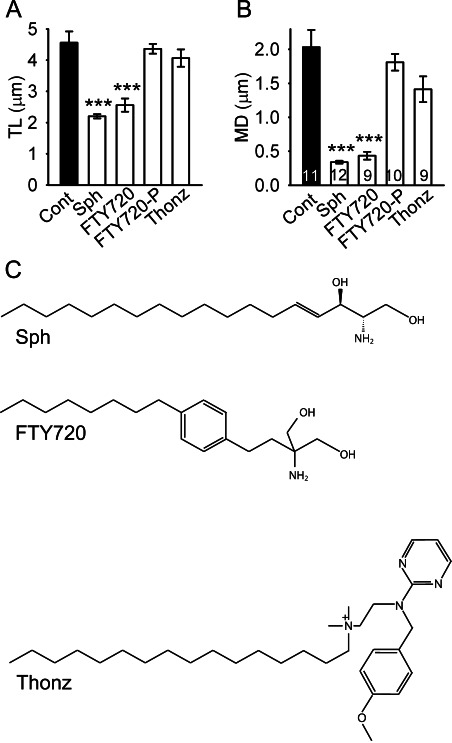
Cell permeable lipids, FTY720 and sphingosine, strongly attenuate peptidergic vesicle mobility. (**A**) The mean (±s.e.) TL and (**B**) MD of vesicles in nontreated control astrocytes (Cont, black bars) and astrocytes treated (white bars) with 10 μM concentration of permeable (sphingosine, Sph; FTY720) or nonpermeable (thonzonium, Thonz; phosphorylated FTY720, FTY720-P) lipids. (**C**) Chemical structures of the lipids used in the mobility assays.

Next, we tested sphingosine—the natural structural analog of FTY720, the phosphorylated FTY720 (FTY720-P) and thonzonium—a membrane impermeable positively charged lipid (Fig. 3C). In cells treated with 10 μM sphingosine the mean vesicle TL (2.20 ± 0.07 μm; *n* = 12) was reduced by ∼52% (*P* < 0.001) when compared with nontreated cells (4.56 ± 0.36 μm; Fig. 3A), similarly to the TL reduction (∼44%) in cells treated with 10 μM FTY720. In contrast, the cell treatment with 10 μM FYT720-P and 10 μM thonzonium did not significantly alter vesicle TL (4.36 ± 0.16 μm, *n* = 10 and 4.07 ± 0.28 μm, *n* = 9, respectively). A similar outcome was also observed in vesicle MD, where 10 μM sphingosine (0.34 ± 0.02 μm) and 10 μM FTY720 (0.44 ± 0.06 μm) reduced the mean vesicle MD by ∼84% and 80%, respectively, when compared with nontreated cells (2.03 ± 0.25 μm; *P* < 0.001), while 10 μM FYT720-P and 10 μM thonzonium did not significantly alter vesicle MD (1.81 ± 0.12 μm and 1.41 ± 0.19 μm, respectively; Fig. 3B). Altogether, our results demonstrate that FTY720 and its membrane permeable natural structural analogue, sphingosine, attenuate vesicle mobility to a similar extent. In contrast, FTY720-P and thonzonium do not significantly affect vesicle mobility (Fig. 3D), likely due to their membrane impermeability.

### FTY720 Inhibits the Mobility of Subplasmalemal and Cytoplasmic Peptidergic Vesicles

**Fig. 4:**
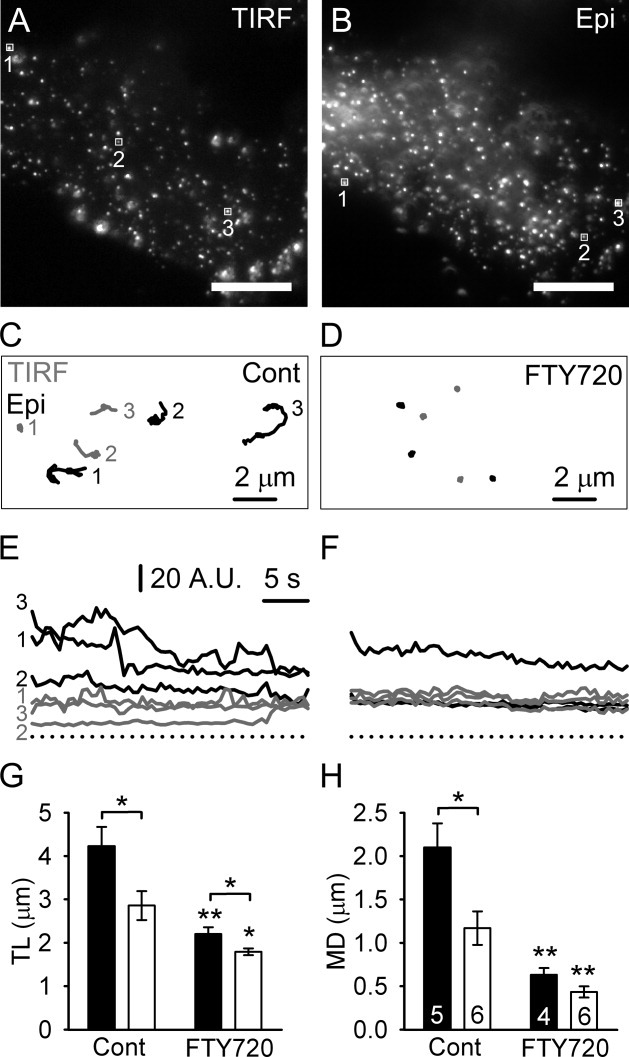
FTY720 similarly attenuates three-dimensional mobility of peptidergic vesicles in two distinct subcellular regions. (**A**) A TIRF and (**B**) an epifluorescence image of the same astrocyte displaying fewer fluorescent vesicles adjacent to the plasma membrane than within the astrocyte cytoplasm. Scale bars, 10 μm. (**C**) The visualization of the selected vesicle tracks (1–3, panels A and B) observed in the *x*-*y* plane in nontreated astrocyte in epifluorescence space (black) and in the TIRF (gray) plane. Selected vesicles tracked in the epifluorescence space were more mobile than the vesicles tracked in the TIRF plane, where one vesicle (1) appeared caged around the initial position, while the other two vesicles (2 and 3) traveled short distance away from their initial positions. (**D**) The vesicle tracks in the *x*–*y* plane in an astrocyte treated with 10 μM FTY720. Note that all vesicles, regardless of their subcellular location, displayed minimal mobility. (**E**) Time-resolved fluorescence intensity profiles of the selected vesicles in nontreated control cell. All three vesicles tracked in the epifluorescence space displaced along the *z*-axis, as suggested by large fluctuations in their fluorescence intensity over time (black). In the TIRF plane (gray) however, the vesicles displayed relatively minor *z*-motions. (**F**) Time-resolved fluorescence intensity of the selected vesicles in the same cell after the treatment with 10 μM FTY720. With the exception of one vesicle in the epifluorescence space (top black trace), all other vesicles did not displace significantly along the *z*-axis. (**G–H**) The quantitative comparison of the mean vesicle TL (**G**) and MD (**H**) obtained in nontreated control cells (Cont) and in cells treated with 10 μM FTY720 within the epifluorescence space (black) and the TIRF plane (white). Note the reduced vesicle mobility within the TIRF plane in nontreated control cells. The treatment of cells with 10 μM FTY720 strongly attenuated vesicle mobility; larger relative attenuation was observed in the epifluorescence plane, while vesicle mobility was arrested the most in the TIRF plane. *P < 0.05, **P < 0.01, vs. respective controls.

To investigate whether FTY720 differently affects vesicle mobility in the subplasmalemal region and deeper in the cytoplasm, we used total internal reflection fluorescence (TIRF) microscopy. Alternate time-lapse acquisition of TIRF (Fig. 4A) and epifluorescence (Fig. 4B) images allowed us to visualize vesicles that were positioned in the proximity (∼150 nm) of the plasma membrane (observed in the epifluorescence and TIRF images), and vesicles that were apparently positioned deeper inside the cells (visualized only in the epifluorescence images). Still, some vesicles visible only in the epifluorescence channel may be potentially close to the plasma membrane due to plasma membrane folds in astrocytes (Pangrsic et al.,^2006^). In general, the vesicles residing deeper in the cytoplasm appeared relatively more mobile than those in the proximity of the plasma membrane (compare black and gray vesicle tracks in Fig. 4C, and the mobility parameters displayed on Fig. 4G,H). Following 10-min treatment with 10 μM FTY720, the mobility of the same vesicles was strongly attenuated regardless of their subcellular location (Fig. 4D,G,H).

In addition to the *x*–*y* mobility of vesicles, we assessed vesicle movements along the *z*-axis by analyzing time-resolved average fluorescence intensity of the selected vesicles. In nontreated astrocytes, the vesicles (1–3, black traces, Fig. 4E) positioned inside the cytoplasm displaced also along the *z*-axis, as suggested by the larger fluctuations in their fluorescence intensity over time (compare black and gray traces in Fig. 4E). With the exception of the vesicle labeled 3 that displayed complex *z*-motion pattern, the other two vesicles positioned in the proximity of the plasma membrane displayed more stable fluorescence intensity, indicating fewer excursions in the *z*-direction (Fig. 4E). Again, as observed before, the cell treatment with 10 μM FTY720 attenuated vesicle movements along the *z*-axis in both subcellular locations; the strong attenuation was indicated by the stable fluorescence intensity of the vesicles tracked inside the cytoplasm and in the proximity of the plasma membrane (Fig. 4F, compare black and gray traces).

Next, we quantitatively compared the vesicle TL and MD obtained in nontreated controls and in cells treated with 10 μM FTY720 by epifluorescence (EPI) and TIRF imaging (Fig. 4G,H). The spontaneous mobility and the mobility attenuated by FTY720 observed in EPI images largely corroborated the mobility observed in confocal images (compare Fig. 2G,H and Fig. 4G,H). However, in the TIRF plane the vesicles in nontreated controls displayed significantly reduced TL and MD (Fig. 4H, Cont black bars) in comparison to epifluorescently imaged vesicles (TL: 2.86 ± 0.33 μm vs. 4.22 ± 0.45 μm, MD: 1.17 ± 0.19 μm vs. 2.10 ± 0.28 μm; *P* < 0.05). Treatment of cells with 10 μM FTY720 caused significant diminution of vesicle mobility, by reducing the mean TL in EPI and TIRF plane by 48% (2.20 ± 0.16 μm, *P* < 0.01) and 37% (1.79 ± 0.08 μm, *P* < 0.05), respectively, while the corresponding MD diminished by 70% (0.63 ± 0.08 μm, *P* < 0.01) and 63% (0.43 ± 0.06 μm, *P* < 0.01) (Fig. 4G, H; FTY720 bars). Altogether, these data indicate that FTY720 strongly attenuates the three-dimensional vesicle mobility irrespective of their subcellular location.

### FTY720 and Sphingosine Inhibit Stimulated Peptide and Glutamate Release

**Fig. 5:**
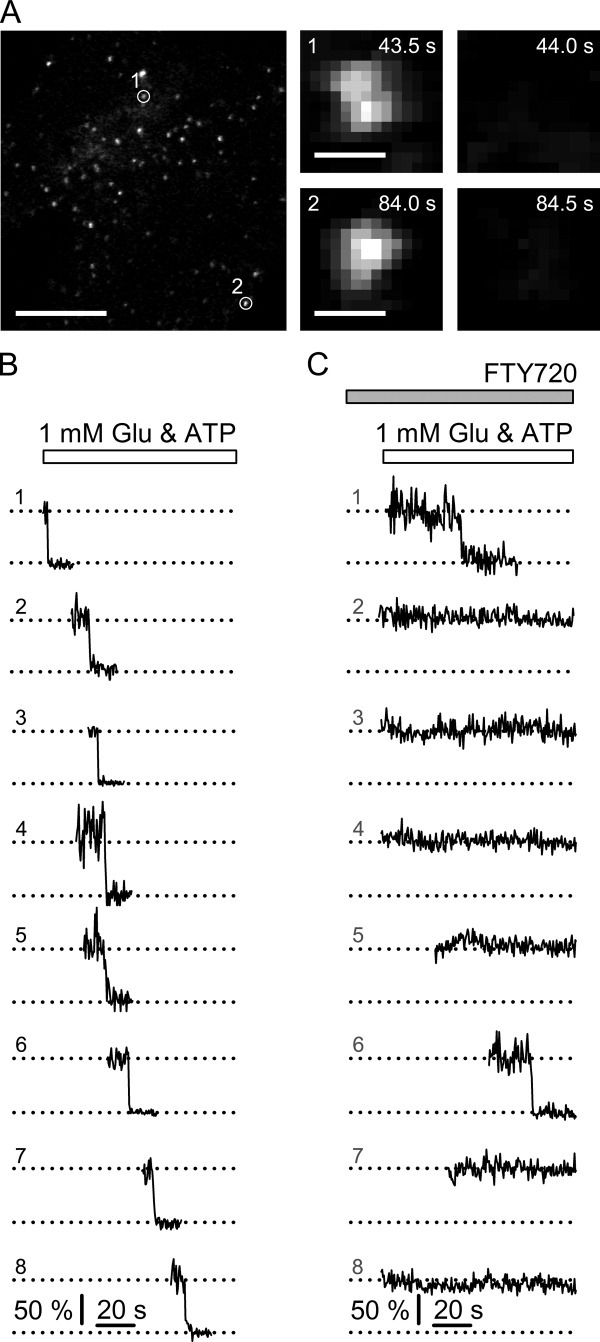
Monitoring the vesicle cargo discharge. (**A**) Confocal image of a nonstimulated astrocyte (left) displaying numerous peptidergic vesicles. Upon stimulation with 1 mM L-Glu and ATP, the vesicles 1 and 2 (encircled) fused and discharged the cargo (insets on the right) as indicated by the rapid and comprehensive diminishment in their fluorescence intensity within 0.5 s; between the two successive time frames. Time marks in top right corners indicate the poststimulation time. Scale bars: 10 μm (left) and 0.5 μm (insets). (**B**) Normalized, time-dependent fluorescence changes assessed in eight selected vesicles (all from panel A, black) display abrupt intensity decline due to complete exocytotic cargo discharge upon stimulation. The dotted lines indicate the minimum and maximum fluorescence levels. The normalized vesicle fluorescence is displayed at the site of fusion, where vesicles moved to from the initially distinct position. (**C**) Normalized time-dependent fluorescence changes assessed in eight selected vesicles (gray) in a cell treated with FTY720. The two vesicles (labeled 1 and 6) display abrupt intensity decline due to stimulated exocytotic cargo discharge, while the fluorescence of other six vesicles persisted at the elevated level throughout the observation time. The time of 1 mM Glu and ATP addition (white) and FTY720 treatment (gray) are indicated.

Subsequently, we examined whether the lipid compounds affect the exocytotic peptide release from astrocytes upon stimulation with L-Glu and ATP, which was shown to trigger a strong and Ca^2+^-dependent exocytotic release of neuropeptide Y from cultured astrocytes (Ramamoorthy and Whim,^2008^). In nontreated cells individual cargo secretion events were observed following stimulation (Fig. 5A), whereby rapid and complete exocytotic discharge of ANP.emd was apparently accomplished within 0.5 s, between the two successive image frames (Fig. 5A, insets right). The normalized, time-dependent fluorescence intensity profile of vesicle cargo discharge (labeled 1 and 2 in Fig. 5A) revealed asynchronous and rapid decrease in vesicle fluorescence, indicating complete cargo release from several vesicles following cell stimulation (Fig. 5B). In contrast, in cells pretreated with 10 μM FTY720 the cargo secretion events were observed only rarely (Fig. 5C).

**Fig. 6:**
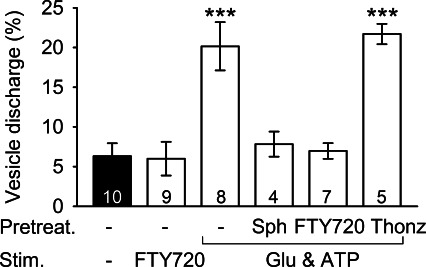
Stimulation-dependent vesicle cargo discharge is attenuated by the astrocyte treatment with FTY720 and sphingosine. (**A**) The histogram displays the mean (±s.e.) percent of vesicles releasing peptides in nonstimulated control cells (black), FTY720 acutely treated cells and cells stimulated with 1 mM L-Glu and ATP, which were either not treated (−) or pretreated with sphingosine (Sph), FTY720, and thonzonium (Thonz). The vesicle counts were obtained prior to and 1 min after the stimulus application. Note that FTY720 did not trigger significant release, while 1 mM L-Glu and ATP triggered substantial release of peptides from nontreated cells and cells pretreated with thonzonium. The pretreatment of cells with sphingosine and FTY720 inhibited stimulated vesicle cargo release. Numbers at the bar bases indicate the number of astrocytes analyzed. ***P < 0.001 versus nonstimulated control.

Further, we statistically evaluated the extent of vesicle cargo discharge by counting vesicles before and 1 minute following the stimulation of astrocytes (*n* = 4–10) that were not treated or pretreated with sphingosine, FTY720, or thonzonium (Fig. 6A). In addition, we examined whether FTY720 itself potentially triggers cargo discharge from astrocytic vesicles. In nontreated cells that were not stimulated afterwards, 6.33% ± 1.62% (*n* = 10) vesicles apparently discharged cargo within 1 min. This was likely due to spontaneous vesicle fusion (Potokar et al.,^2008^), although possible defocusing inevitably associated with confocal microscopy may account for this as well (Kreft et al.,^2005^; Stenovec et al.,^2004^). Acute application of 20 μM FYT720 to astrocytes resulted only in a minor apparent discharge from vesicles (3.69% ± 1.08% within 1 min and 6.00% ± 2.12% within 10 min; *n* = 9). Stimulation of nontreated cells with L-Glu and ATP triggered a substantial (*P* < 0.001) increase in vesicle cargo discharge (20.15% ± 3.04%; *n* = 8). In contrast, the treatment of cells with sphingosine or FTY720 apparently prevented stimulated cargo discharge triggered by L-Glu and ATP; treatment with sphingosine or FTY720 resulted in 7.83% ± 1.58% (*n* = 4) and 6.97% ± 0.99% (*n* = 7) responses upon stimulation, respectively. Interestingly, the treatment of cells with thonzonium did not affect stimulated exocytotic release (21.70% ± 1.28%; *n* = 5), as the vesicle discharge triggered by L-Glu and ATP was similar to the one observed in nontreated cells.

Finally, we examined whether short-term 10 μM FTY720 treatment affects astrocytic glutamate release measured fluorimetrically (Innocenti et al.,^2000^). In confluent cultures, elevations in NADH fluorescence were observed after cell stimulation with 1 mM ATP (data not shown). The statistical comparison of peak amplitudes obtained in nontreated (*n* = 20) and FTY720 treated cells (*n* = 30) indicated a ∼42% reduction of glutamate release monitored as the regional reduction in the NADH peak fluorescence, which was 4.05 ± 0.32 A.U. (*n* = 60) vs. 2.35±0.19 A.U. (*n* = 90) in nontreated versus FTY720-treated cells (*P* < 0.001), respectively.

## DISCUSSION

A number of recent studies highlighted the importance of lipid metabolism in the regulation of exocytosis. Sphingosine was shown to have multiple and even opposing roles in regulated exocytosis in neurons and in neuroendocrine cells (Camoletto et al.,^2009^; Darios et al.,^2009^), but it was never examined in astrocytes, which also participate in the control of brain circuit function (Verkhratsky and Parpura,^2010^). Here we examined the effect of sphingosine and the structurally similar molecules—FTY720, FTY720-P, and thonzonium, on the mobility of vesicles and vesicle cargo discharge from cultured astrocytes by the confocal and TIRF microscopies. We show here for the first time that small, cell-permeable lipid molecules, sphingosine and FTY720, strongly and dose-dependently attenuate the mobility of peptidergic vesicles, as well as the mobility of glutamatergic vesicles and endosomes/lysosomes. This effect appears not to be confined only to astrocytes, since peptidergic vesicle mobility was attenuated also in rat lactotrophs and mouse 3T3-L1 cells.

Sphingosine affects the molecular interactions between the proteins establishing the SNARE complex (Camoletto et al.,^2009^; Darios et al.,^2009^), therefore the observed attenuation of vesicle mobility may involve the SNARE proteins, which take part in intracellular vesicle traffic and fusion steps along the secretory pathway (Jahn and Scheller,^2006^). By using TIRF microscopy we show that in basal conditions, where only spontaneous mobility of vesicles was monitored, vesicles proximal to the plasma membrane of astrocytes appear less mobile in comparison to those in the deeper cytoplasm, consistent with the previous reports, suggesting a system of tethers or a heterogeneous matrix that limits the vesicle motion (Johns et al.,^2001^; Oheim and Stühmer,^2000^). Cell treatment with FTY720 attenuated the mobility of vesicles regardless of their subcellular location. The observed effects of sphingosine-like lipids may originate from more common modification of vesicle ability to translocate along the cytoskeleton (Potokar et al.,^2007^). The cytoskeleton plays an essential role in vesicle dynamics, as distinct motor proteins propel secretory vesicles along microtubules and actin filaments (Goldstein and Yang,^2000^; Soldati and Schliwa,^2006^). When individual cytoskeletal components were pharmacologically depolymerized (Potokar et al.,^2007^), the mobility of astrocytic peptidergic vesicles was substantially reduced. Therefore, it appears likely that FTY720 and sphingosine may directly or indirectly inhibit vesicle mobility via the cytoskeleton-dependent mechanism.

An additional factor that may be involved in the regulation of vesicle mobility is protein kinase C (PKC), which is inhibited by both, sphingosine (Bazzi and Nelsestuen,^1987^; Hannun et al.,^1986^) and FTY720 (Sensken and Gräler,^2010^). It has been reported that PKC activation enhances exocytosis in neurons (Stevens and Sullivan,^1998^) and chromaffin cells (Gillis et al.,^1996^) by increasing the readily releasable pool of secretory vesicles, while in PC12 cells the enhanced exocytosis is due to increased rate of vesicle delivery to the plasma membrane in a PKC-dependent manner (Zhang et al.,^2008^). A number of proteins involved in different aspects of exocytosis have been identified as PKC substrates, among them SNAP-25, where PKC phosphorylation of SNAP-25 (Shimazaki et al.,^1996^) potentiates vesicle recruitment (Nagy et al.,^2002^) and sensitizes Ca^2+^-dependent vesicle release (Yang et al.,^2007^). Interestingly, PKC activation in astrocytes leads to phosphorylation of SNAP-23 and a reduction in regulated exocytosis of a peptide hormone (Yasuda et al.,^2011^). It is possible that this contrasting PKC effect on astrocytic secretion might be due SNAP-23, rather than SNAP-25, expression in these glial cells. Another target of PKC is Munc-18 (Fujita et al.,^1996^), a protein associated to the SNARE complex, which plays a role in vesicle docking and fusion (Jorgacevski et al.,^2011^); Munc 18-1 is expressed in astrocytes (Zhang et al.,^2004^). Its phosphorylation enhances vesicle priming/docking (Fujita et al.,^1996^) and potentiates vesicle pool replenishment likely via the inhibition of the association of Munc-18 with syntaxin and the consequent increase of free syntaxin for SNARE complex formation (Nili et al.,^2006^). Furthermore, the activation of PKC causes the disassembly of peripheral F-actin cytoskeleton and thus facilities vesicle access to the plasma membrane (Gil et al.,^2000^), likely via phosphorylation of myristoylated alanine-rich C kinase substrate (MARCKS) (Park et al.,^2006^) or other actin-filament-associated proteins (Larsson,^2006^). Although the aforementioned implications of the PKC in the exocytotic process mostly address its activation, it is conceivable to assume that PKC inhibition by sphingosine and FTY720 would, in contrast, result in decreased rate of vesicle delivery to the plasma membrane, mediated either by nonphosphorylation of the proteins involved in vesicle traffic or by preventing the disassembly of peripheral F-actin cytoskeleton.

An interesting finding is that FTY720 and sphingosine inhibit stimulation-dependent vesicle peptide discharge from astrocytes. This is in contrast to neurons and neuroendocrine cells where sphingosine upregulated stimulated exocytotic cargo releases by facilitating the SNARE complex assembly (Darios et al.,^2009^). However, our finding corroborates the results of Williams et al. (2009), where sphingosine inhibited calcium dependent fusion of insulin granules in Min6B1 cells (transformed mouse β-cells) by acting as an amphipathic weak base that neutralizes the electrostatic surface potential of phospholipid membranes, important for the electrostatic interaction with the highly conserved positively charged carboxy terminal juxtamembrane domains of membrane SNARE protein syntaxin and the vesicle SNARE protein VAMP (Montal,^1999^). It was also reported that the formation of the SNARE complex may be attenuated due to the sphingosine-mediated enhanced interaction between Munc-18 with syntaxin (Camoletto et al.,^2009^), an event upstream of SNARE complex assembly (Jorgacevski et al.,^2011^), which may explain the reduced vesicle docking (Camoletto et al.,^2009^). These interactions are consistent with multiple and even opposing effects of sphingosine on regulated exocytosis, and therefore require further clarification. While sphingosine likely facilitates docking of the vesicles adjacent to the plasma membrane, it may arrest the delivery of the vesicles arriving from the cell interior to the plasma membrane. Therefore, heterogeneous effects of sphingosine may depend on the cell type specific vesicle pools that contribute to the vesicle membrane fusion and the subsequent vesicle cargo release upon stimulation. While in neurons the readily releasable pool of vesicles that are docked at the active zones at presynaptic plasma membrane and primed for fusion, account for the rapid initial burst of exocytotic release, astrocytes appear not to have such a defined arrangement of vesicles, and the kinetics of the Ca^2+^-regulated exocytosis in astrocytes is ∼2 orders of magnitude slower (Kreft et al.,^2004^) than in neurons (Heidelberger et al.,^1994^; Kreft et al.,^2003^). This further implies that the rate of vesicle delivery from the cell interior to the plasma membrane significantly determines the efficiency of exocytotic release in astrocytes. The measurements of astrocytic glutamate release provide experimental support for this scenario.

An important question is whether the FTY720 effects described here can occur in patients taking this drug. While the therapeutic dose of the drug is expected to yield submicromolar range in the blood plasma (Meno-Tetang et al.,^2006^), it is possible that micromolar concentration of FTY720 can be attained at least adjacent to the tissue hydrophobic pools of the drug. It was shown (Foster et al.,^2007^) that FTY720 accumulates in the white matter in the CNS to exceed blood concentrations several fold. In these areas, the local concentration of FTY720 may reach concentrations that affect vesicle mobility in astrocytes and impact their secretory activity. Astrocytes were considered the major source of eicosanoids (prostaglandins, prostacyclins, thromboxanes, and leukotrienes)—proinflammatory signaling molecules in CNS that are released via ATP-dependent mechanism (Murphy et al.,^1988^). FTY720 may inhibit their release (Foster et al.,^2007^); thus, the observed effects may be part of the therapeutic effectiveness of FTY720 in patients with multiple sclerosis. This possibility has to be investigated further, by considering the requirement for prolonged exposure to FTY720 to impair the formation of eicosanoids.

In conclusion, the results of this study have shown that the sphingosine-like molecule FTY720 plays a regulatory role in astrocytic vesicle dynamics, affecting regulated exocytosis of peptides, which was also observed in other cell types studied. We propose that the attenuation of vesicle mobility hinders the delivery of vesicles to the fusion competent sites at the plasma membrane, which may play a role in the therapy of multiple sclerosis and other immunoneurological disorders.
